# Deoxycholic Acid Could Induce Apoptosis and Trigger Gastric Carcinogenesis on Gastric Epithelial Cells by Quantitative Proteomic Analysis

**DOI:** 10.1155/2016/9638963

**Published:** 2016-12-14

**Authors:** Yanyan Shi, Ying Wei, Ting Zhang, Jing Zhang, Ye Wang, Shigang Ding

**Affiliations:** ^1^Research Center of Clinical Epidemiology, Peking University Third Hospital, Beijing 100191, China; ^2^Department of Gastroenterology, Peking University Third Hospital, Beijing 100191, China; ^3^Department of Microbiology, Peking University Health Science Center, Beijing 100191, China

## Abstract

*Background.* Pathologic duodenogastric reflux can induce or aggravate gastritis because of the presence of bile acids. Bile reflux has been generally considered to be associated with intestinal metaplasia and gastric cancer. However, the pathogenic mechanisms of the effects of bile acids on gastric mucosa are still unknown.* Methods.* To explore the mechanisms by which bile acids induce gastric mucosal lesions, we examined cell apoptosis in the gastric epithelial cell line GES-1 and investigated the changes in protein profiles of GES-1 cells in response to a bile acid deoxycholic acid using a proteomics approach. Changes in the profiles of the differently expressed proteins were analyzed using the DAVID and STRING programs.* Results.* We found apoptosis was significantly induced in GES-1 cells by deoxycholic acid. Using liquid chromatographic/tandem mass spectrometric (LC-MS/MS) methods, 134 upregulated proteins and 214 downregulated proteins were identified in the bile acid treated GES-1 cells. Bioinformatics analysis revealed the interactions and signaling networks of these differentially expressed proteins.* Conclusion.* These findings may improve the understanding of the molecular mechanisms underlying the pathogenicity of bile acids on gastric mucosa.

## 1. Introduction

Reflux of bile is one of the main etiological factors in the pathophysiological processes leading to gastric mucosal lesions in patients with chronic gastritis [1]. Bile reflux gastritis (BRG) has been recognized to be a chemical gastropathy due to excessive duodenogastric reflux. Physiological duodenogastric refluxate does not contain bile acids but only contains HCO_3_^−^ and IgA, which might have protective functions for gastric mucosa [2]. Nevertheless, pathologic duodenogastric reflux can be induced by many factors, such as abnormalities in pyloric anatomic structure and antropyloric and duodenal dysmotility, with continuous bile acids secretion. Pathologic duodenogastric reflux can induce or aggravate gastritis because of the presence of bile acids, and high concentration of bile acids may play a critical role in the induction of intestinal metaplasia (IM) in the stomach. Furthermore, bile reflux is also believed to function as an initiator of gastric carcinogenesis [3].

Some studies have found that bile acids and other contents of the duodenum act synergistically in the development of chronic gastritis with gastric acid and* Helicobacter pylori *infection [4, 5]. Apoptosis and redox reactions have been reported to be associated with bile acid-induced gastritis [6, 7]. Gastric IM induced by bile reflux is considered to be a precancerous gastric adenocarcinomal lesion and is associated with the induction of cyclooxygenase-2 (COX-2). However, the exact pathogenic mechanisms by which bile acids affect the gastric mucosa are still not clear.

In this study, we assessed the influence of a bile acid deoxycholic acid on the gastric epithelial cell line GES-1, and proteomics analysis was used to identify the biological processes and molecular pathways through which deoxycholic acid exerts its pathogenic effects on gastric mucosa.

## 2. Materials and Methods

### 2.1. Cells, Culture Conditions, and Deoxycholic Acid Treatment

GES-1 cells and AGS cells were both cultured in RPMI1640 growth medium supplemented with 10% (v/v) fetal bovine serum (Hyclone, Logan, UT, USA) at 37°C in a humidified incubator in 5% (v/v) CO_2_. A 10 mM deoxycholic acid (Sigma, St. Louis, MO, USA) stock solution was prepared in PBS and was incubated in a water bath at 37°C for 30 min before each use.

Cells were seeded in growth medium one day before deoxycholic acid treatment. For coculturing of cells and deoxycholic acid, cells were rinsed once with PBS before fresh growth medium was added. Concentrations of 200 *μ*M and 400 *μ*M of deoxycholic acid were used in the preliminary experiment (shown in supplementary figure in Supplementary Material available online at http://dx.doi.org/10.1155/2016/9638963), and 400 *μ*M was chosen in the following studies. Deoxycholic acid was added to the cell medium at the final concentration of 400 *μ*M, and the cells were maintained under normal growth conditions for 10 h. Untreated GES-1 cells were used as controls.

### 2.2. Apoptosis Assay

GES-1 cells and AGS cells were both seeded in 6-well plates (at a density of 1.2 × 10^5^ cells). After incubating the cells with or without deoxycholic acid for 10 h, an Annexin V-FITC/PI double-staining Apoptosis Detection Kit (Becton Dickinson, Franklin Lakes, NJ, USA) was used to label the cells according to the manufacturer's instructions. Untreated GES-1 cells were used as negative controls. Cells were washed with cold PBS, and 200 *μ*L of the Annexin V-Binding Buffer was added. After the cells were stained with 10 *μ*L of FITC-labeled Annexin V and 5 *μ*L of PI, they were immediately analyzed by flow cytometry.

### 2.3. Protein Extraction and SDS-PAGE

GES-1 cells cultured with or without deoxycholic acid were harvested. For protein extraction, cells were suspended in cell lysis buffer containing a protease inhibitor mixture and shaken on ice for 30 min. The cell lysate was centrifuged at 15,000 ×g at 4°C for 10 min, and the supernatant was collected. The total protein concentration was measured by the Bradford method using a BCA Protein Assay Kit (Thermo Fisher Scientific, Waltham, MA, USA). Proteins (200 *μ*g) were separated by 15% (w/v) SDS-PAGE. The gels were then stained with Coomassie brilliant blue G-250 (Bio-Rad Laboratories, Hercules, CA, USA) to examine differences between the total proteins of GES-1 cells with and without deoxycholic acid.

### 2.4. NanoLC-MS/MS and Data Analysis

In this study, a highly specific and sensitive liquid chromatographic/tandem mass spectrometric (LC-MS/MS) method was used to identify differently expressed proteins in GES-1 cells cultured with or without bile acids. The gels were divided into 15 equal pieces according to the proteins' molecular weights. Proteins were then digested with trypsin and the peptides applied to an EASY-nLC system (Proxeon Biosystem, Thermo Fisher Scientific) coupled online to an ESI-LTQ-OrbitrapVelos mass spectrometer (Thermo Fisher Scientific) mostly as described previously [8]. Peptides were eluted through a trap column and an analytical column packed with C-18 ReproSil 3 *μ*m resin using a gradient from 100% phase A (0.1% formic acid in water) to 35% phase B (0.1% formic acid in acetonitrile) for 150 min. Mass spectra were acquired in a positive mode using the data-dependent automatic (DDA) survey MS scan and tandem mass spectra (MS/MS) acquisition. The DDA survey scan was of the* m/z* range 350–2000 and resolution 60,000 with a target value of 1 × 10^−6^ ions. The survey scan was followed by MS/MS of the 15 most intense ions in the LTQ using the collision-induced dissociation (CID), and previously fragmented ions were dynamically excluded for 30 s. Raw data were searched against the Swiss-Prot human proteome database using MaxQuant software (version 1.2.2.5). Searches were performed with the following parameters: tryptic hydrolysis, two missed cleavages, oxidation of methionine as variable modification, carbamidomethylation as fixed modification, and peptide tolerance of 10 ppm. Search results were subsequently processed/filtered through the Search Engine Processor tool [9] using a 1% false discovery rate (FDR).

Identified proteins were BLAST searched against the NCBI nonredundant database. Ontological analysis of the differentially expressed proteins was performed using the search tool DAVID (http://david.abcc.ncifcrf.gov/). STRING (http://string.embl.de) was used as a database for predicted signaling networks and protein interactions as previously described [10].

### 2.5. Western Blot Analysis

Protein samples of the two groups were performed by western blotting to validate the differentially expressed proteins. For protein extraction, cells were suspended in cell lysis buffer containing a protease inhibitor mixture and shaken on ice for 30 min. The cell lysate was centrifuged 15,000 ×g at 4°C for 10 min, and the supernatant was collected. The total protein concentration was measured by the Bradford method using a BCA Protein Assay kit. Proteins (70 mg) were separated on 12% (w/v) SDS-PAGE gels and electrophoretically transferred onto PVDF membranes. The membranes were blocked in 5% (w/v) fat-free milk in Tris-buffered saline, 0.5% (v/v) Tween-20, at room temperature for 1 h and incubated overnight at 4°C with antibodies against SOS1 (Flarebio, China; 1 : 500), PTK2 (Flarebio; 1 : 500), ATP12P (Flarebio; 1 : 500), H2AFY (Flarebio; 1 : 500), and *α*-tubulin (MBL, Japan; 1 : 2000). After three washes in PBS supplemented with 0.1% (v/v) Tween-20 for 15 min, the membranes were incubated with a secondary antibody, goat anti-Rabbit IRDye 680 or goat anti-Mouse IRDye 800CW (LICOR; 1 : 5000), for 1 h at room temperature. Proteins were identified by scanning the membranes using the Odyssey Imager (LI-COR Biosciences).

### 2.6. Statistical Analysis

The differences between two groups were analyzed using Student's* t*-test. Data are presented as the mean ± SD of three independent experiments. All statistical analyses were performed using SPSS 21.0 software. *P* values < 0.05 were considered statistically significant.

## 3. Results

### 3.1. Deoxycholic Acid Induced Apoptosis in GES-1 Cells

**Figure 1:**
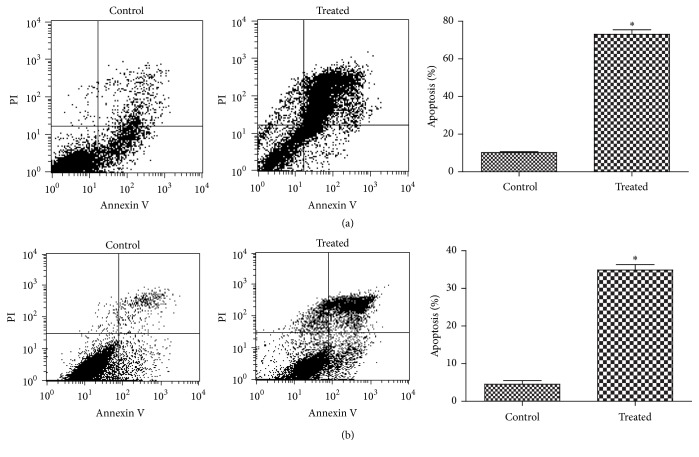
(a) A representative experiment of Annexin V-FITC/PI double-staining of GES-1 cells at 10 h of treatment is shown. The proportion of Annexin V positive cells (AV+: apoptotic cells) in the control and treated groups were, respectively, 10.25% and 73.11%. (b) A representative experiment of Annexin V-FITC/PI double-staining of AGS cells at 10 h of treatment is shown. The proportion of Annexin V positive cells in the control and treated groups were, respectively, 4.54% and 34.9%. Data are presented as the mean ± SD of three independent experiments. ^*∗*^When compared with the control, *P* values < 0.05 were considered statistically significant.

An apoptosis detection kit was used to detect apoptosis induced by a 400 *μ*M final concentration of deoxycholic acid in GES-1 cells. The results (Figure 1(a)) indicated that deoxycholic acid induced apoptosis in GES-1 cells (*P* < 0.05). AGS cells were measured in the same way as GES-1 cells (Figure 1(b)).

### 3.2. Protein Purification and Identification

The proteome of the GES-1 cells was profiled using SDS-PAGE with Coomassie brilliant blue staining and LC-MS/MS. If the ratio of a protein was >2.0 or <0.5 in the cells treated with bile acid relative to negative control cells, the protein was considered to be differentially expressed. In total, 348 differentially expressed proteins were identified, including 214 downregulated proteins and 134 upregulated proteins (shown in supplementary data). The ratios of 14 different proteins were >5.0 or <0.2, including 6 downregulated proteins (Table 1) and 8 upregulated proteins (Table 2), indicating that they are highly regulated and of particular interest.

### 3.3. Cluster Analysis of the Bile Acid-Regulated Proteins

**Figure 2:**
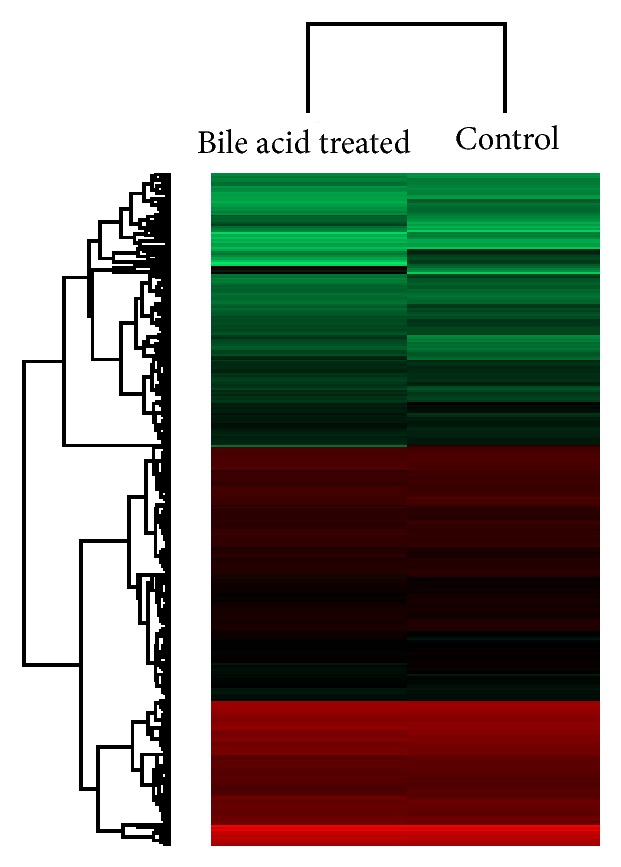
The expression levels of 348 dysregulated proteins are shown in a heatmap. The red-colored clusters represent upregulated proteins and the green-colored clusters represent downregulated proteins.

A heatmap was constructed from the data obtained for the 348 differentially expressed proteins. The clustering analysis of the differentially expressed proteins in the bile acid-treated cells provides evidence that the genes encoding these proteins are regulated by deoxycholic acid (Figure 2).

### 3.4. Functional Classification and Enrichment of the Bile Acid-Regulated Proteins

**Figure 3:**
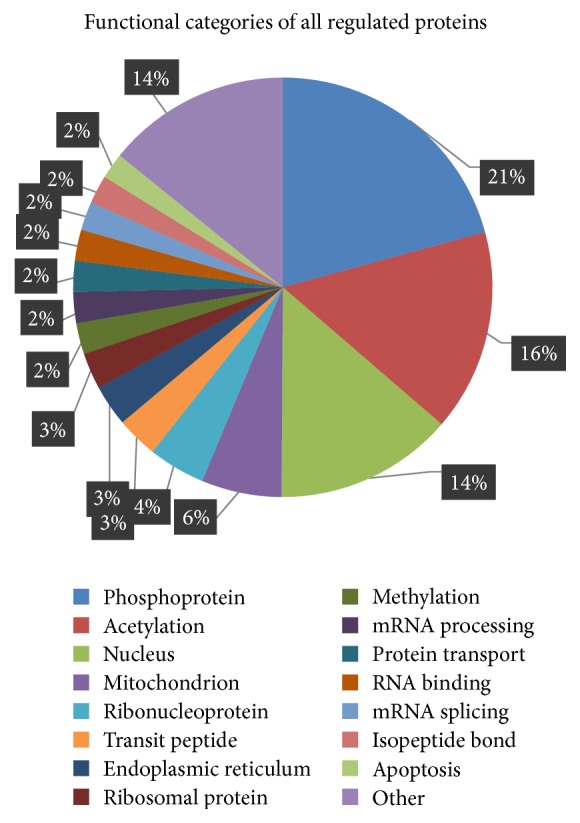
The functional categories of the dysregulated proteins are shown in a pie. It shows that phosphoprotein and acetylation proteins contributed the most proportion in the dysregulated proteins.

**Figure 4:**
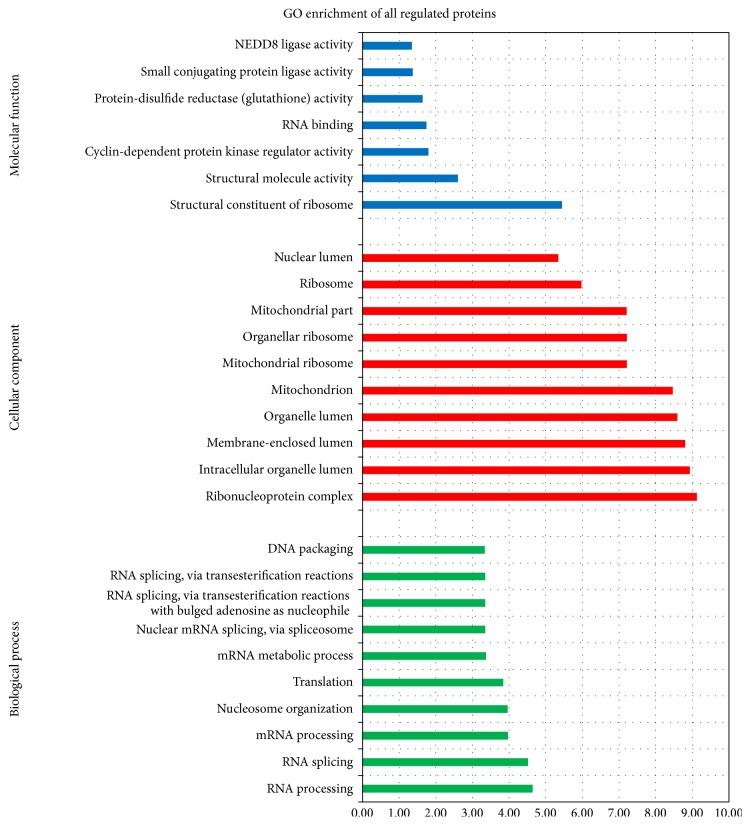
The 348 dysregulated proteins were analyzed according to gene ontology terms. All of the dysregulated proteins were analyzed by searching the DAVID database, *P* < 0.05. The histograms show the top 10 counts for molecular function, cellular component, and biological process.

The identified differentially expressed proteins were classified into the functional categories shown in Figure 3, and proteins involved in phosphorylation and acetylation were the highest proportions. More interestingly, it is shown that mitochondrial proteins account for approximately 6 percent of the identified proteins, which may play roles in cell apoptosis. To further elucidate the biological processes affected by bile acid, these dysregulated proteins were annotated to the DAVID database for enrichment analysis in terms of biological process, cellular component, and molecular function (Figure 4).

### 3.5. Analysis of the Signaling Network

**Figure 5:**
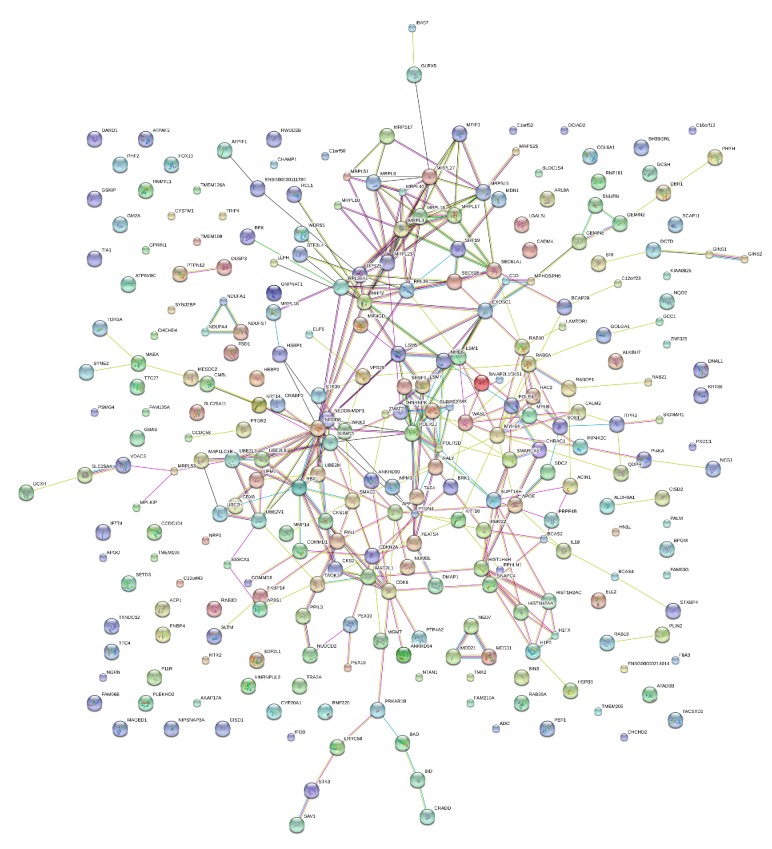
The differentially expressed proteins were used to search the STRING database to predict their protein-protein interactions in the deoxycholic acid treated cells. In the network, the nodes are the proteins and the lines represent the predicted functional associations. The number of lines indicates the strength of the predicted functional interactions of the proteins.

The differentially expressed proteins after bile acid treatment were then searched against the STRING database, and 341 proteins had matches in the database. The chosen confidence level (STRING score) was 0.4. A merged network is shown in Figure 5. The substantial significant functions of the proteins in the network were RNA binding, structural constituent of ribosome, nucleic acid binding, and protein kinase regulator activity. ACIN1, AKAP17A, Clorf52, and CID are important proteins in RNA binding and nucleic acid binding. MRPL10, MRPL16, and MRPL17 are important proteins in structural constituent of ribosome. ANKRD54, CALM2, and CDKN2A are important proteins in protein kinase regulator activity. The program predicted associations for a particular group of proteins.

### 3.6. Verification of Four Identified Proteins

**Figure 6:**
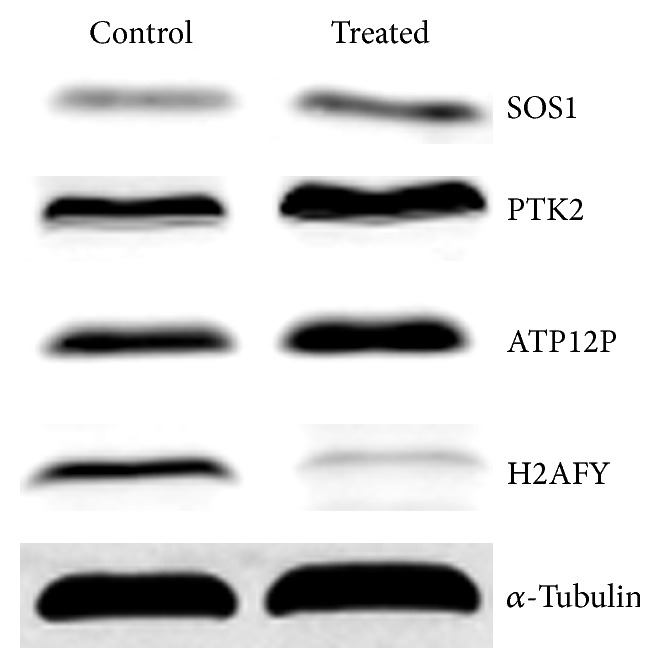
Protein expression analysis for four pivotal proteins including SOS1, PTK2, ATP12P, and H2AFY in GES-1 cells by Western blot. GES-1 cells were treated with deoxycholic acid. Western blot analyses were performed using whole extracts of cells treated with deoxycholic acid. *α*-Tubulin was used as an endogenous reference.

In support of the above results, western blot analysis was conducted to monitor changes in the level of four identified proteins implicated in DNA repair and cell cycle (Figure 6). In comparison with the negative control, core histone macro-H2A.1 (H2AFY) was downregulated after being treated by deoxycholic acid. Son of sevenless homolog 1 (SOS1), focal adhesion kinase 1 (PTK2), and ATP synthase mitochondrial F1 complex assembly factor 2 (ATP12P) were upregulated. These were consistent with the results of quantitative proteomic analysis.

## 4. Discussion

There is a strong association between the concentration of bile acid in the duodenogastric refluxate and the degree of gastroesophageal reflux disease (GERD), and bile reflux is a main risk factor for Barrett's esophagus [11, 12]. Bile acid exposure can exacerbate gastric mucosal lesions such as those caused by active or chronic inflammation. Moreover, length of time of bile acid exposure correlates with the severity of pathological changes in the gastric mucosa [1]. In addition, bile acid directly induces intestinal metaplasia and progression to neoplasia of the esophagus and stomach [5, 13, 14].

Although bile acid is thought to be critical in the pathogenesis of gastric mucosal diseases, the mechanisms by which bile acids induce transformation in the stomach are still not clear [15]. Compared with studies of the stomach mucosa, there have been many more studies focusing on mechanisms of esophageal mucosal diseases induced by bile acid. Recent studies indicate that immune responses and/or signaling pathways that regulate cell proliferation or cell phenotypes can cause damage or metaplasia of esophageal epithelial cells [16]. In ex vivo/in vitro studies, bile acids stimulate esophageal cells to produce inflammatory mediators (e.g., IL-8 and COX-2) and cause oxidative stress, DNA damage, and apoptosis. Bile acids also induce squamous cells to change their gene expression pattern to resemble intestinal-type cells and cause Barrett's cells to increase expression of intestinal-type genes [17]. However, in the gastric mucosa, there are glandular epithelial cells, which are different from squamous cells in esophageal mucosa.

Whether pathogenesis mechanisms of bile acids in the stomach are the same as in esophagus has not been confirmed. The mechanisms of gastric cellular death induced by bile acids remain controversial. In this study, we used proteomics analysis of a human gastric mucosal cell line treated with a bile acid deoxycholic acid to investigate the mechanisms. It has been reported that deoxycholic acid amounted to about 27% of total bile acids. The presence of mainly free secondary and primary bile acids may contribute to the high incidence of cancer in the gastric remnant observed after Billroth operations and reflux of bile exists [18]. Unconjugated bile acids deoxycholic acid and chenodeoxycholic acid could dramatically affect esophageal cells during the development of Barrett's esophagus, especially deoxycholic acid [19]. Some researchers used deoxycholic acid to establish chronic gastritis animal models [20]. These may indicate deoxycholic acid is one of the most cytotoxic bile acids and has been used previously to study bile or bile acids. Based on this consideration, we used deoxycholic acid in this study. Previous studies reported minimal cellular injury in response to 100 *μ*M or 200 *μ*M deoxycholic acid exposure [21, 22], and higher concentrations of deoxycholic acid (500 *μ*M–1 mM) have been used to study injury to gastric cells and hepatocytes [23]. Moreover, the deoxycholic acid concentration in human gastric remnants after distal gastric resections is approximately 370 *μ*M. Hence, we employed concentrations of 200 *μ*M and 400 *μ*M for 5 h, 10 h, and 24 h in the preliminary experiments (shown in Supplementary Figure  S) and chose 400 *μ*M for 10 h finally. Our results showed that deoxycholic acid could induce apoptosis in gastric mucosa cell lines GES-1 and AGS, and the latter cell line AGS was used to avoid the cell-specific effects. Some studies have implicated a necrotic pathway in the effects of deoxycholic acid on gastric cells [22, 24, 25], whereas others have proposed that the effects are due to apoptosis [6, 26]. The present study indicated that apoptosis might be a major mechanism of deoxycholic acid-induced gastric mucosal cell death.

However, the mechanisms for the inflammation- and cancer-induction effects of bile acids on the gastric mucosa have not yet been determined. Proteomics analysis in this study revealed a total of 348 differently expressed proteins, which were found to be involved in diverse biological processes, including RNA binding, structural constituent of the ribosome, nucleic acid binding, and protein kinase regulator activity. Some of the identified proteins have been reported to be involved in inflammation and neoplasia. Core histone macro-H2A.1 and E3 ubiquitin-protein ligase UHRF1 were downregulated, which has been reported to be related with the obstruction of DNA repair and nucleotide metabolism and thus induction of genetic mutations or epigenetic defects [27, 28]. Proteins involved in cell proliferation, including the membrane associated proteins n-chimaerin [29], son of sevenless homolog 1 [30, 31], and focal adhesion kinase 1 [32] were upregulated. In contrast, Bcl2, an antagonist of cell death, was downregulated. Our observations suggested that cell proliferation was triggered after bile acid treatment. The cells might undergo genomic events leading to foveolar hyperplasia, which is one of the important histopathological features of reflux gastritis and is essential for tumorigenesis [33]. ATP synthase mitochondrial F1 complex assembly factor 2 was also upregulated, suggesting enhanced oxidative phosphorylation and accelerated energy metabolism [34, 35], which is consistent with an increase in cell proliferation. Besides, some studies have reported that when cell proliferation is stimulated, cell apoptosis is meanwhile easier to be induced. Oncogene activation could meanwhile make cells more sensitive to apoptosis. The procedures of cell proliferation and apoptosis are coupled with each other [36–38]. Bile reflux is believed to function as an initiator of gastric carcinogenesis [39, 40]. Our results imply that bile acids would drive the development and progression of bile reflux gastritis and even gastric cancer.

Collectively, our results suggest that bile reflux is one of the primary factors in the pathogenesis of gastric mucosal lesions and should be the focus of further attention. Apoptosis of gastric mucosal cells could be induced by deoxycholic acid. Proteomics analysis identified the differently expressed proteins in GES-1 cells treated with deoxycholic acid. These findings improve our understanding of the molecular mechanisms underlying the effects of deoxycholic acid on gastric mucosa cells. Future researches should be performed in patients to further investigate the clinical relations of the proteins identified from this vitro study.

## 5. Conclusions

In summary, our integrated analysis revealed a profile of differently expressed proteins in deoxycholic acid-treated gastric mucosal cells. Moreover, the results of function enrichment analysis revealed that some of these proteins may have biological functions related to the development of gastric mucosal diseases induced by deoxycholic acid, including in RNA splicing, macromolecular complex subunit organization, and nucleosome organization. The signaling network analysis may contribute to further understanding the underlying regulatory mechanisms of bile acid-induced gastric lesions, including gastric cancer.

## Supplementary Material

GES-1 cells were exposed to deoxycholate acid (200 μM and 400 μM) for 5 h, 10 h, 24 h. Results are presented as mean ± s.d. of three independent experiments. Cell apoptosis is concentration- and time- dependent after treated with deoxycholate acid.

## Figures and Tables

**Table 1:** Downregulated proteins in GES-1 cells treated with deoxycholic acid (ratio < 0.2).

Number	Protein name	Ratio^*∗*^	p*I*	Nominal mass	Protein function
1	Core histone macro-H2A.1	0.176	9.8	39617	Plays central roles in transcription regulation, DNA repair, DNA replication, and chromosomal stability
2	Cytospin-B	0.126	6.29	118585	Not clear
3	CREB-regulated transcription coactivator-3	0.133	6.35	66959	Transcriptional coactivator for CREB1 which activates transcription through both consensus and variant cAMP response element (CRE) sites
4	Bcl-2 antagonist of cell death	0.196	6.6	18392	Promotes cell death and successfully competes for the binding to Bcl-X (L), Bcl-2, and Bcl-W, thereby affecting the heterodimerization of these proteins with BAX
5	Vacuolar-sorting protein SNF8	0.139	6.2	28864	Component of the endosomal sorting complex required for transport II (ESCRT-II), which is required for multivesicular body (MVB) formation and sorting of endosomal cargo proteins into MVBs
6	E3 ubiquitin-protein ligase UHRF1	0.169	7.66	89813	Multidomain protein that acts as a key epigenetic regulator by bridging DNA methylation and chromatin modification

^*∗*^Ratio of the specific value of expression intensity of the protein in cells treated with or without deoxycholic acid.

**Table 2:** Upregulated proteins in GES-1 cells treated with deoxycholic acid (ratio > 5).

Number	Protein name	Ratio^*∗*^	p*I*	Nominal mass	Protein function
1	Cell cycle control protein 50A	5.917	8.81	40683	Not clear
2	UPF0428 protein CXorf56	11.628	8.94	25624	Not clear
3	ATP synthase mitochondrial F1 complex assembly factor 2	7.092	6.62	32772	May play a role in the assembly of the F1 component of the mitochondrial ATP synthase (ATPase)
4	snRNA-activating protein complex subunit 4	8.547	8.51	159432	Part of the SNAPc complex required for the transcription of both RNA polymerase II and polymerase III small-nuclear RNA genes
5	N-chimaerin	6.098	6.51	53172	GTPase-activating protein for p21-rac and a phorbol ester receptor, involved in the assembly of neuronal locomotor circuits as a direct effector of EPHA4 in axon guidance
6	Son of sevenless homolog 1	5.155	6.38	152464	Promotes the exchange of Ras-bound GDP by GTP, catalytic component of a trimeric complex that participates in transduction of signals from Ras to Rac by promoting the Rac-specific guanine nucleotide exchange factor (GEF) activity
7	Focal adhesion kinase 1	5.952	6.19	119233	Nonreceptor protein-tyrosine kinase that plays an essential role in regulating cell migration, adhesion, spreading, reorganization of the actin cytoskeleton, formation and disassembly of focal adhesions and cell protrusions, cell cycle progression, cell proliferation, and apoptosis
8	Protein transport protein Sec61 subunit alpha isoform 1	7.143	8.3	52264	Plays a crucial role in the insertion of secretory and membrane polypeptides into the ER

^*∗*^Ratio of the specific value of expression intensity of the protein in cells treated with or without deoxycholic acid.
