# Supplementary material for: Deoxycholic Acid Could Induce Apoptosis and Trigger Gastric Carcinogenesis on Gastric Epithelial Cells by Quantitative Proteomic Analysis
Source: Gastroenterol Res Pract. 2016 Dec 14;2016:9638963. doi: 10.1155/2016/9638963 (PMC5192292; doi:10.1155/2016/9638963)

Supplementary figure legend:

Concentration and time dependence of apoptosis of treated with deoxycholate acid. GES-1 cells were exposed to deoxycholate acid (200 μM and 400 μM) for 5 h, 10 h, 24 h. Results are presented as mean ± s.d. of three independent experiments.

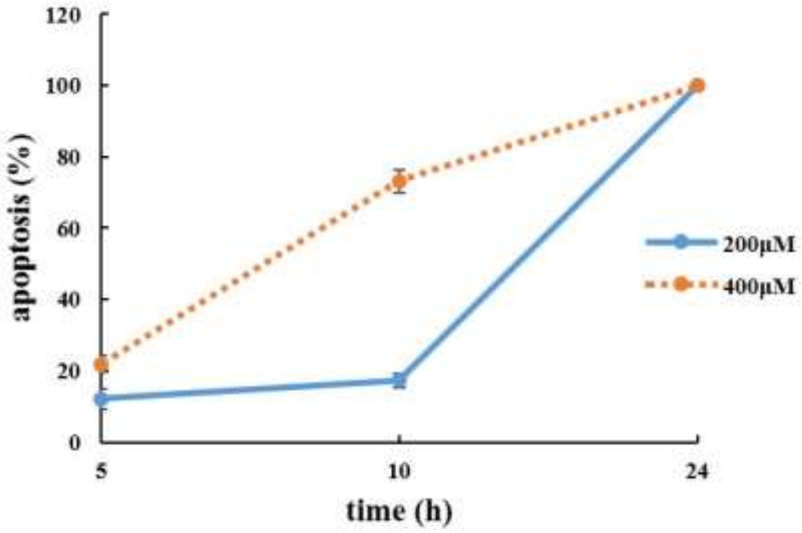

Supplement: Supplementary file 1 — GES-1 cells were exposed to deoxycholate acid (200 μM and 400 μM) for 5 h, 10 h, 24 h. Results are presented as mean ± s.d. of three independent experiments. Cell apoptosis is concentration- and time- dependent after treated with deoxycholate acid. [file 9638963.f1.pdf]
